# What can Parents' Self‐report of Reading Difficulties Tell Us about Their Children's Emergent Literacy at School Entry?

**DOI:** 10.1002/dys.1571

**Published:** 2017-09-18

**Authors:** Zahra Esmaeeli, Kjersti Lundetræ, Fiona E. Kyle

**Affiliations:** ^1^ Norwegian Reading Centre University of Stavanger Stavanger Norway; ^2^ Division of Language and Communication Science City, University of London London UK

**Keywords:** emergent literacy, family risk, reading difficulties, dyslexia, home literacy environment, parental self‐report of reading difficulties

## Abstract

Research has linked family risk (FR) of reading difficulties (RD) with children's difficulties in emergent literacy development. This study is the first to apply parents' self‐report of RD as a proxy for FR in a large sample (n = 1171) in order to test group differences in children's emergent literacy. Emergent literacy, the home literacy environment and children's interest in literacy and letters were compared across different groups of FR children around the school entry. The FR children performed lower in emergent literacy compared with not‐FR children. Furthermore, when comparing FR children with one parent reporting RD and children with both parents reporting RD, moderate group differences were found in Emergent Literacy. Finally, parents' self‐report of RD was a significant contributor of emergent literacy after controlling for the home literacy environment, children's gender, their interest in literacy and letters, months in kindergarten, vocabulary and parents' education. Our findings suggest that schools should monitor the reading development of children with parents self‐reporting RD closely – especially if both parents self‐report RD. © 2017 The Authors. *Dyslexia* published by John Wiley & Sons Ltd.

## Abstract 2

**Key Messages**

- The principal implication is the value of screening for reading difficulties with the simple but valid tool ‘parents' self‐report of reading difficulties (RD)’ in preschool years.
- If the parents had themselves faced RD, their children are more likely to experience difficulties in developing emergent literacy.
- The risk of difficulties in emergent literacy is higher when both parents have a history of RD.
- Parents, especially with self‐reported RD, should be advised about the role of home literacy environment in the development of their children's emergent literacy.
- Families with both parents self‐reporting RD have the fewest children's books at home.
- Family risk children reported less interest in letters than not‐family risk children; parents should be advised to discuss letters and sounds during shared reading.
- Schools should monitor the reading development of children with parents self‐reporting RD closely – especially if both parents self‐report RD.

Reading difficulties (RD) refer to specific difficulties in acquiring reading, writing and basic reading subskills such as word identification and phonological decoding and are not because of extraneous factors such as general learning difficulties, sensory acuity deficits, socioeconomic disadvantage and similar factors (Vellutino, Fletcher, Snowling, & Scanlon, 2004). RD can run in families (Pennington & Olson, 2005), and having a parent or a sibling with RD places the child at high risk for RD, known as *family risk* (FR) (Elbro, Borstrom, & Petersen, 1998; Gallagher, Frith, & Snowling, 2000; Pennington & Lefly, 2001; Scarborough, 1990; Snowling, Gallagher, & Frith, 2003; Snowling, Muter, & Carroll, 2007). Previous research has consistently documented lower emergent literacy for children at FR for RD compared with children without FR (not‐FR) in the preschool years (Carroll & Snowling, 2004; Elbro & Petersen, 2004; Torppa *et al*., 2007; Torppa *et al*., 2012; van Bergen, van der Leij, & de Jong, 2014).

Whitehurst and Lonigan (2001) refer to emergent literacy as early skills, knowledge and attitudes related to print, which originate and develop throughout the preschool years. The National Early Literacy Panel conducted a meta‐analysis and reported that letter knowledge, concepts about print, oral language (such as vocabulary) and phonological sensitivity (e.g. phonemic awareness) are the components of emergent literacy that are most predictive of children's later reading success (Lonigan, Schatschneider, & Westberg, 2009). It is now clear that early individual differences in emergent literacy can strongly predict both later reading achievement (Pinto, Bigozzi, Vezzani, & Tarchi, 2016; Scarborough, 2001; Snow, Burns, & Griffin, 1998; Wagner & Torgesen, 1987) and RD (Bigozzi, Tarchi, Pezzica, & Pinto, 2016; Elbro *et al*., 1998; Pennington *et al*., 2012; Pennington & Lefly, 2001; Snowling & Hulme, 2013).

In a recent meta‐analysis, Snowling and Melby‐Lervåg (2016) provide a comprehensive review of FR studies that have compared FR children with not‐FR on emergent literacy. They report group differences in favour of not‐FR children on measures of letter knowledge (*d* = 0.47), phoneme awareness (*d* = 0.56), vocabulary (*d* = 0.65), rhyme (*d* = 0.90), rapid automatized naming (RAN) (*d* = 0.61) and verbal short‐term memory (STM) (*d* = 0.45) at preschool age. They found that the reported effect sizes differed between studies depending upon the language context, choice of assessments, age of the groups and, most pertinently, the type of criteria used to identify poor readers: the prevalence was lower for studies that used more conservative criteria. Overall, the meta‐analysis found that FR children perform significantly poorer in emergent literacy compared with not‐FR. However, no studies have investigated within‐group differences of FR children in order to compare emergent literacy between FR children who had only one parent with RD (FR‐one) and FR children who had both parents with RD (FR‐both). On the basis of genetic studies, we know that having both parents with RD may put the child at higher risk for RD rather than having one parent with RD (Wolff & Melngailis, 1994).

Prior research on FR for RD has tended to include a direct measure of parents' literacy skills in addition to parents' self‐report of RD (Carroll & Snowling, 2004; Snowling & Melby‐Lervåg, 2016; Torppa *et al*., 2012). This was partly because the validity and reliability of self‐report of RD had not been documented and also the majority of FR studies had relatively small sample sizes, allowing for parents' literacy skills to be directly assessed. However, a growing body of FR research is currently based on parents' self‐report of RD, and it is now considered to be both a valid and reliable measure (Leavett, Nash, & Snowling, 2014; Lefly & Pennington, 2000; Snowling, Dawes, Nash, & Hulme, 2012) and a time‐saving instrument for estimating RD in adults (Snowling *et al*., 2012). For example, Leavett *et al*. (2014) found that adults who self‐reported as having RD had significantly poorer skills in word reading and spelling.

In the current study, we use parents' self‐report of RD as a single proxy for FR status. The main aim is to investigate group differences in emergent literacy between not‐FR and FR children and to explore possible within‐group differences in FR children with one parent reporting RD (FR‐one) and FR children with both parents reporting RD (FR‐both) while controlling for background variables such as home literacy environment (HLE), parental education, children's gender, months in kindergarten and their oral language skills (vocabulary).

The HLE, which includes home literacy activities such as shared reading, children's access to print and parents' own reading interest and habits, is another important factor for children's emergent literacy (Lonigan, Burgess, & Anthony, 2000; Whitehurst & Lonigan, 2001). However, several FR studies have reported that the frequency of child–parent shared reading at home did not significantly differ between the FR and not‐FR groups, even though parents of FR children were less active readers themselves than the not‐FR parents (Elbro *et al*., 1998; Lyytinen *et al*., 2004; Torppa *et al*., 2006, 2007). These studies were conducted in Finland and Denmark, in which the parental/maternal education level did not significantly differ between FR and not‐FR groups. Equivalent maternal education might be the reason for the non‐significant differences in HLE aspects between these groups. In contrast, research in England has shown that parents with RD exposed their children to fewer shared‐reading activities compared with not‐FR parents (Dilnot, Hamilton, Maughan, & Snowling, 2017; Hamilton, Hayiou‐Thomas, Hulme, & Snowling, 2016; Scarborough, Dobrich, & Hager, 1991).

In line with these results, van Bergen, van Zuijen, Bishop and de Jong (2016) have recently found that HLE correlated significantly with children's reading fluency. In addition, paternal and maternal reading fluency explained independent and similarly large proportions of variance in children's reading fluency: together, parental reading fluency explained 17% of variance in children's reading fluency. In another study, moderate correlations were found between children and parents' reading skills: ~0.35 for fathers and ~0.50 for mothers (Van Bergen *et al*., 2012). In addition to HLE, children's interest in literacy has been found to be strongly associated with the development of emergent literacy in FR children (Torppa *et al*., 2007). Because the development of emergent literacy, like other developmental skills, seems to be a multifactorial process involving both genetic and environmental factors, individual differences in children's emergent literacy are likely to be associated with FR status (which in turn is related to the variation in their parents' reading skills) and parental education besides HLE and children's interest in literacy. Specifically, the second aim of the present study is to compare different aspects of HLE and children's interest in literacy in groups of children differing in FR status based upon their parents' self‐report of RD. The final aim is to explore the associations between FR status and children's emergent literacy before the onset of reading instruction while controlling for different aspects of HLE, children's interest in literacy, years in kindergarten, gender, vocabulary and parental education. On the basis of the existing literature, the research questions for the current study and our hypotheses are as follows:

1. Can parents' self‐report of RD identify between‐group and within‐group differences in emergent literacy skills? We expect that children identified by parents' self‐report of RD will display lower skills in emergent literacy compared with not‐FR children and that children with both parents self‐reporting RD have even lower emergent literacy than children with only one parent self‐reporting RD.
2. Is there an effect of FR status on the HLE and children's interest in literacy? We expect that not‐FR families will report the richest HLE and that the FR‐both will obtain the lowest score in aspects of HLE. However, group differences are not expected in children's interest in literacy before formal instruction of reading because some earlier studies have not reported such differences (Torppa *et al*., 2006, 2007).
3. Does FR status predict emergent literacy after controlling for HLE, children's interest in literacy, years in kindergarten, gender, vocabulary and parental education? We hypothesize that parents' self‐report of RD is a unique predictor of children's emergent literacy after controlling for background variables including HLE, children's interest in literacy, years in kindergarten, gender, vocabulary and parental education.

## Methods

### Participants

The sample was selected from an ongoing Norwegian large‐scale longitudinal project (On Track), with 1171 participating 6‐year‐old first graders. The majority of parents (97.7%) gave their consent for participation. The study was reviewed and approved by the Norwegian Social Science Data Service, a third‐party ethical oversight agency. Based on exclusion of children with Norwegian as second language (*n* = 193), bilingual (*n* = 83), hearing problems (*n* = 28), dropout (*n* = 29) or parents who did not answer whether they had experienced RD or did not know about biological parents' reading skills (*n* = 74), the sample for the present study was 821 children living in two municipalities in the southwest of Norway. In Norway, formal reading instruction starts in grade 1, and 96.7% of primary‐school students are enrolled in public schools (i.e. non‐private) (Utdanningsdirektoratet, 2016). Norwegian is a semi‐transparent orthography that is more regular than English and less regular than Finnish. Parents' educational level was used as a proxy for socioeconomic status because previous research has shown that in Norway, parental level of education is a stronger predictor of educational outcomes than parents' income (Løken, 2010).

#### Family risk

Each participating school held a welcome meeting for parents before the new children started at school. The ‘On Track’ team presented the project, including information about RD at these meetings. Parents were invited to take part in the study and received an information brochure and a parental consent form. At the beginning of the first grade, participating parents answered a questionnaire relating to demographics, HLE, familial risk of RD, the student's language background and his or her health. FR status was obtained through the question ‘has the child's biological mother and/or father experienced “reading and writing difficulties”?’, and the response options were ‘yes’, ‘no’ or ‘don't know’. ‘Reading and writing difficulties’ is a familiar term in Norway, relating to specific problems with word recognition and spelling. The term is frequently used in schools and the media, and it was discussed at the welcome meeting.

While 634 children had no parent self‐reporting a history of RD (not‐FR), 187 children had one or both parents with a self‐reported history of RD (FR). In addition, the FR children were divided into two groups: FR‐one, children with only one parent self‐reporting RD (*n* = 165), and FR‐both, children with both parents self‐reporting RD (*n* = 22).

Attendance in kindergarten did not statistically differ between FR (98.9%) and not‐FR (99.5%) groups. FR and not‐FR groups did differ in parental educational level (mothers' educational level: *X* ^2^ (*N* = 819, 2) = 39.96, *p* < 0.001; fathers' educational level: *X* ^2^ (*N* = 817, 2) = 33.91, *p* < 0.001. In fact, both mothers and fathers in the not‐FR group had significantly higher levels of education than the FR‐one and the FR‐both groups. However, group differences in parental education between FR‐one and FR‐both did not reach significance. Table 1 presents the children's characteristics by group.

**Table 1:** Children's characteristic by group based on the parents' self‐report of RD

				FR		
			Not‐FR	FR (all)	FR‐one parent	FR‐both parents
Sample size			634	187	165	22
Age (M, sd)			6.22 (0.28)	6.22 (0.30)	6.23 (0.30)	6.11 (0.26)
Gender: boys (%)			47.30	52.40	53.90	40.90
Years in kindergarten (M, sd)			4.61 (0.71)	4.45 (0.94)	4.41 (0.98)	4.78 (0.45)
Parental level of education (%)^a^	Mothers*	Low	3.00	9.10	8.50	13.60
		Medium	23.20	40.60	38.80	54.4
		High	73.70	50.30	52.70	31.80
	Fathers*	Low	3.80	9.10	7.30	22.70
		Medium	33.80	50.80	51.50	45.50
		High	62.30	38.50	39.40	31.80

RD, reading difficulties; FR, family risk; FR‐one, FR children who had only one parent with RD; FR‐both, FR children who had both parents with RD.

a Parental level of education: low, primary school; medium, upper secondary school; high, university/college.

* *p* < 0.001.

### Procedure and Measures

The test battery was individually administered and scored on a digital tablet between 2 and 5 weeks after school started in grade 1. The testing was carried out by a team of 18 trained testers who were experts within the field of reading education and individual testing. The parents answered a questionnaire on demographics, HLE, FR based on parents' self‐report of RD and the child's interest in literacy and letters and their language background and health. The parents' questionnaire was the only paper–pencil‐based measure in the study.

#### Emergent literacy measures

*Letter knowledge* consisted of a 15‐item multiple‐choice test. The child was asked to listen to a pre‐recorded letter sound on the tablet and respond by pressing on one of the four touch‐screen letters. The reliability (Cronbach's alpha) was 0.85.

In *first phoneme isolation*, the tablet screen showed a picture, and the examiner pointed to the picture, named it and asked the child about the first sound of that word. The oral response of the child was scored and recorded on the tablet by the examiner. This task contained eight items and Cronbach's *α* = 0.92.

The *blending* task required the child to blend a set of separately pronounced phonemes into the corresponding whole word. The test had eight items of increasing difficulty and was automatically discontinued after two subsequent errors. In each item, four pictures appeared on the screen, and the task was pre‐recorded: ‘Here you see a picture of /ri/–/rips/–/ris/ and /ring/ (ride, red current, rice, ring, in English). Listen carefully and touch the picture that goes with: /r/−/i/−/s/ (presented phoneme‐by‐phoneme, one per second)’ (Cronbach's *α* = 0.86).

*Vocabulary* was tested with an abridged version (20 out of 40 words) of the Norwegian vocabulary test (Størksen, Ellingsen, Tvedt, & Idsøe, 2013). A picture appeared on the screen, and the child was asked to name it. Reliability (Cronbach's alpha) for 20 items in the present sample was 0.83, which is consistent with the 40 items in the standardized sample (0.84).

#### Reading‐related measures

*Rapid automatized naming* (RAN) included naming familiar objects presented repeatedly in random order. The examiner practised the task with the child and made sure that the child knew the name of each object and understood the procedure of the task. The pictured objects were *sun*, *car*, *plane*, *house*, *fish* and *ball*, which are all monosyllabic words in Norwegian. There were four rows of five stimuli in each matrix and two trials. The child was asked to name each item as quickly and accurately as possible from the left to the right and from the top to the bottom. Time to complete the task (in seconds) and naming errors were recorded.

*Short‐term memory* was measured with Digit Span Forward from Wechsler Intelligence Scales for Children – III (Wechsler, 1991). The examiner read aloud one digit per second, and the student's responses were scored on the tablet.

*Word reading* included eight words ranging from easy to difficult representing a variety of letters and letter sequences (VC, CV, CVC, VCV, CVC, CVCC, and CVCCV). The words appeared on the screen one at a time. The child was asked to read the word aloud. Cronbach's alpha was 0.92.

*Spelling* involved 10 words with a variety of phonemes and phonemes sequences ranging from easy to difficult (CV, VC, VCV, VCC, CVC, CVCV, CVCC, and CVCVC). There was a practice trial in which the examiner wrote down the word while sounding out each letter and asked the child to do the same. For the test items, each target word was introduced in a short sentence; then, the examiner repeated the target word and encouraged the child to write the word (e.g. ‘Father has a blue hat. Write /hat/.’). Cronbach's alpha was 0.93.

#### Home literacy environment and children's interest in literacy and letters

On the basis of previous research, different components of the HLE were measured via parents' questionnaires (Dilnot *et al*., 2017; Hamilton *et al*., 2016; Niklas & Schneider, 2013; Skwarchuk, Sowinski, & LeFevre, 2014; Torppa *et al*., 2007).

1. The child's access to print was assessed with the following items: (a) How many children's books do you have at home? (1 to 5 (*none* to *more than 40 books*)). (b) How old was the child when you first started reading to her or him? (1 to 5 (*Never read to the child* to *before the age of 2*)).
2. Literacy‐related activities included the four following questions: (a) How often do you read to the child? (b) How often does the child watch TV? (c) How often does the child play TV/computer/tablet/mobile games? (d) How often do you visit a library with the child? (1 to 5 (*never* to *several times a week*)).
3. Parents' reading habits were assessed by questions regarding how often they read (a) books and (b) magazines for themselves (1 to 5 (*never* to *several times a week*)). Parents' own reading interest was assessed by the item ‘I only read if I have to’ (1 to 4 (*completely disagree* to *completely agree*)).
4. *Child's interest in literacy and letters* was assessed through the items (a) My child often asks to be read to and (b) My child takes an interest in letters (1 (*completely disagree*) to 4 (*completely agree*)).

## Results

Those variables displaying skewness greater or lower than +/−1 and kurtosis greater or lower than +/−2 were subjected to square‐root transformation to enable parametric statistical techniques to be applied. Where there was a negative skew, distributions were reflected before square‐root transformation was applied. Skewedness for letter knowledge was (−1.34), and RAN was (1.25). However, kurtosis's were between +/−2 in all measures. The transformed variables were used in the inferential analyses, whereas the results here are presented for the raw data because the results were the same for both raw and transformed variables.

### Group Differences between FR and Not‐FR Children According to Parents' Self‐report of RD

Confirmatory factor analysis was conducted on the data from the questionnaire using Mplus program. The overall goodness of fit (Brown, 2014) indicated that our HLE model fits the data well: (root mean square error of approximation (RMSEA) = 0.05; comparative fit index (CFI) = 0.94; Tucker‐Lewis index (TLI) = 0.91). Factor loading estimates revealed that the indicators were strongly related to their purported factors and components of HLE (range of *R* ^2^s = 0.52–0.78). Factor scores for three components of HLE access to print, literacy‐related activities, parents own reading habits, as well as one factor for child's interest in literacy and letter, were calculated. To adjust for multiple comparisons and reduce type I error, ANOVA followed by Bonferroni tests (Tabachnick, Fidell, & Osterlind, 2001) were run to investigate group differences in emergent literacy, the three HLE factors and children's interest in literacy. Table 2 presents the group means for the emergent literacy items and the HLE factor scores. Not‐FR children scored significantly higher than the FR children in all measures of emergent literacy: letter knowledge (*d* = 0.47), first phoneme isolation (*d* = 0.62), blending (*d* = 0.52), vocabulary (*d* = 0.24), word reading (*d* = 0.63), spelling (*d* = 0.55), RAN (*d* = 0.33) and STM (*d* = 0.26).

**Table 2:** Mean differences (*sd*) and effect sizes in *emergent literacy* and HLE between not‐FR and FR groups based on parents' self‐report of RD

		Not‐FR (*N* = 634)	FR (*N* = 187)	*t* (df)	Cohen's *d*
Children's outcomes in emergent literacy and early reading and spelling at the onset of formal reading instruction	Letter knowledge (0–15)	12.52 (*3.19*)	10.96 (*3.42*)	5.50* (288.81)	0.47
	First phoneme isolation (0–8)	5.99 (*2.70*)	4.19 (*3.10*)	7.12* (274.68)	0.62
	Blending (0–8)	3.90 (*2.66*)	2.85 (*2.40*)	5.10* (332.11)	0.52
	RAN	59.35 (*14.23*)	64.66 (*17.57*)	3.78* (261.84)	0.33
	STM (*digit Span*)	5.78 (*1.55*)	5.38 (*1.58*)	3.02* (819)	0.26
	Vocabulary (0–20)	13.66 (*3.24*)	12.85 (*3.39*)	3.36* (819)	0.24
	Word reading (0–8)	3.96 (*3.04*)	2.22 (*2.45*)	8.01* (370.52)	0.63
	Spelling (0–8)	2.88 (*3.00*)	1.36 (*2.44*)	7.07* (367.48)	0.55
Aspects of HLE and children's interest in literacy	Access to print^a^	0.05 (*0.64*)	−0.17 (*0.78*)	3.46* (260.38)	0.31
	Literacy activities^a^	0.04 (*0.69*)	−.15 (*0.69*)	3.33* (803)	0.27
	Parents reading habits^a^	0.07 (*0.68*)	−0.21 (*0.94*)	3.58* (240.29)	0.34
	Child's interest in literacy and letters^a^	0.04 (*0.52*)	−0.11 (*0.60*)	2.96* (270.88)	0.27

HLE, home literacy environment; FR, family risk; RD, reading difficulties; RAN, rapid automatized naming; STM, short‐term memory.

a Factor scores.

* Significant at *p* < 0.001.

Not‐FR families scored significantly higher than FR families in all HLE components: access to print, literacy‐related activities and parents own reading habits (Table 2). Less interest in literacy and letters was also reported for the FR children compared with the not‐FR children.

### Group Differences among FR‐one parent, FR‐both parents and not‐FR children According to Parents' Self‐report of RD

To find out whether group differences could also be observed within the FR group, the FR children were divided into two groups according to their parents' self‐report of RD: FR‐one and FR‐both. Two *MANOVAs* were computed for emergent literacy (*F*(16, 1618) = 0.89, *p* < 0.001; partial η2 = 0.05) and the three HLE factors and children's interest in literacy and letters (*F*(8, 1570) = 0.94, *p* < 0.001; partial η2 = 0.03). Table 3 shows the mean (emergent literacy items) and factor scores (HLE) for each group and the results of post hoc tests followed up by Dunnett's T3 tests, which is more robust for unequal variances and unequal group sizes while maintaining control over the significance level across multiple tests (Tabachnick *et al*., 2001).

**Table 3:** Mean differences (*sd*) and effect sizes in *emergent literacy* and HLE between not‐FR, FR‐one and FR‐both based on parents' self‐report of RD

		Not‐FR	FR		*f* test	Cohen's *d* a
			FR‐one	FR‐both		
Children's outcomes in emergent literacy and early reading and spelling at the onset of formal reading instruction	Letter knowledge (0–15)	12.52 (*3.19*)	11.256 (*3.23*)	8.77 (*4.08*)	22.7, *p* < 0.001; partial η2 = 0.05	Not‐FR and FR‐one*: 0.39
						Not‐FR and FR‐both*: 1.02
						**FR‐one and FR‐both** * **: 0.67**
	First phoneme isolation (0–8)	5.99 (*2.70*)	4.38 (*3.04*)	2.73 (*3.19*)	33.50, *p* < 0.001; partial η2 = 0.08	Not‐FR and FR‐one*: 0.56
						Not‐FR and FR‐both*: 1.1
						**FR‐one and FR‐both** ** **: 0.53**
	Blending (0–8)	3.90 (*2.66*)	2.90 (*2.44*)	2.45 (*2.04*)	81.27, *p* < 0.001; partial η2 = 0.03	Not‐FR and FR‐one*: 0.40
						Not‐FR and FR‐both**: 0.61
						**FR‐one and FR‐both: 0.20**
	RAN	59.35 (*14.23*)	64.07 (*16.85*)	69.12 (*22.24*)	10.10, *p* < 0.001; partial η2 = 0.02	Not‐FR and FR‐one*: 0.30
						Not‐FR and FR‐both*: 0.53
						**FR‐one and FR‐both: 0.27**
	STM (*digit Span*)	5.78 (*1.55*)	5.46 (*1.60*)	4.77 (*1.27*)	6.60, *p* < 0.001; partial η2 = 0.02	Not‐FR and FR‐one: 0.20
						Not‐FR and FR‐both*: 0.71
						**FR‐one and FR‐both: 0.48**
	Vocabulary (0–20)	13.66 (*3.24*)	13.13 (*3.28*)	10.73 (*3.51*)	11.21, *p* < 0.001; partial η2 = 0.03	Not‐FR and FR‐one: 0.17
						Not‐FR and FR‐both*: 0.87
						**FR‐one and FR‐both** * **: 0.71**
	Word reading (0–8)	3.96 (*3.04*)	2.28 (*2.44*)	1.77 (*2.58*)	26.16, *p* < 0.001; partial η2 = 0.06)	Not‐FR and FR‐one*: 0.61
						Not‐FR and FR‐both*: 0.78
						**FR‐one and FR‐both: 0.21**
	Spelling (0–8)	2.88 (*3.00*)	1.40 (*2.48*)	1.05 (*2.19*)	20.27, *p* < 0.001; partial η2 = 0.05	Not‐FR and FR‐one*: 0.54
						Not‐FR and FR‐both**: 0.70
						**FR‐one and FR‐both: 0.15**
Aspects of HLE and children's interest in literacy	Access to print^b^	0.05 (*0.64*)	−0.06 (*0.69*)	−0.74 (*0.90*)	14.70, *p* < 0.001; partial η2 = 0.04	Not‐FR and FR‐one: 0.16
						Not‐FR and FR‐both*: 1.01
						**FR‐one and FR‐both** * **: 0.85**
	Literacy activities^b^	0.04 (*0.69*)	−0.14 (*0.68*)	−0.35 (*0.77*)	7.32, *p* < 0.001; partial η2 = 0.02	Not‐FR and FR‐one**: 0.26
						Not‐FR and FR‐both**: 0.53
						**FR‐one and FR‐both: 0.29**
	Parents' reading habits^b^	0.07 (*0.68*)	−0.16 (*0.94*)	−0.58 (*0.94*)	12.50, *p* < 0.001; partial η2 = 0.03	Not‐FR and FR‐one*: 0.28
						Not‐FR and FR‐both*: 0.79
						**FR‐one and FR**‐**both** ** **: 0.45**
	Child's interest in literacy and letters^b^	0.04 (*0.52*)	−0.10 (*0.59*)	−0.33 (*0.71*)	7.97, *p* < 0.001; partial η2 = 0.02	Not‐FR and FR‐one**: 0.25
						Not‐FR and FR‐both*: 0.59
						**FR**‐**one and FR**‐**both: 0.35**

HLE, home literacy environment; FR, family risk; FR‐one, FR children who had only one parent with RD; FR‐both, FR children who had both parents with RD; RD, reading difficulties; RAN, rapid automatized naming; STM, short‐term memory.

a Effect sizes were reported for both significant and non‐significant group differences.

b Factor scores.

* Significant at *p* < 0.001.

** Significant at *p* < 0.04.

There was a significant main effect of group for all measures of emergent literacy. The hypothesis that children in the FR‐both group would perform at the lowest level in emergent literacy followed by FR‐one children, who in turn would be poorer than the not‐FR group, was supported for letter knowledge and first phoneme isolation. The group differences between FR‐one and FR‐both children were relatively large for letter knowledge (*d* = 0.67), first phoneme isolation (*d* = 0.53) and vocabulary (*d =* 0.71). FR‐one children scored significantly higher than FR‐both children in vocabulary, but there was no difference between FR‐one and not‐FR groups. For measures of blending, word reading, spelling and RAN, both FR‐one and FR‐both performed significantly lower in comparison with not‐FR children; however, differences between FR‐one and FR‐both did not reach significance. Moreover, the only group difference in STM (*d* = 0.71) was found between not‐FR and FR‐both. Generally, our data suggest that FR children with both parents self‐reporting as RD have more severe deficits in certain measures of emergent literacy: letter knowledge, first phoneme isolation and vocabulary.

Turning to the HLE factors and the children's interest in literacy and letters, the ‘parents' own reading habits’ factor was the only measure on which the not‐FR group scored significantly higher than FR‐one, who in turn obtained significantly higher scores than FR‐both. For ‘access to print’, FR‐one children did not differ from not‐FR children, whereas a significant large effect was found between not‐FR and FR‐both groups (*d* = 1.01) and within the FR groups (*d* = 0.85). In contrast, for the component of ‘literacy‐related activities’, significant effects were found between not‐FR and FR‐one children (*d* = 0.26) and between not‐FR and FR‐both children (*d* = 0.53), but no significant difference was found within the FR group for this component. Similarly, not‐FR children scored higher than both FR‐one (*d* = 0.25) and FR‐both (*d* = 0.59) on the ‘interest in literacy and letters’ factor; however, FR‐one and FR‐both did not significantly differ.

### The Role of FR Status (based on Parents' Self‐report of RD) in Determining Emergent Literacy at the Beginning of Formal Reading Instruction

To assess the relative importance of FR status (using parents' self‐report of RD) on emergent literacy, a hierarchical regression analysis was conducted. Letter knowledge, first phoneme isolation and blending were used to make a factor score for emergent literacy. Table 4 shows the correlations of FR status with the other measures of the study. Taking the sample as a whole, FR status was negatively correlated with the HLE factors, children's interest in literacy and letters and the emergent literacy factor.

**Table 4:** Correlations of family risk (FR) status, parental education, the aspects of home literacy environment, children's interest in literacy and letters, emergent literacy and vocabulary in the whole sample

	1.	2.	3.	4.	5.
1. FR status					
2. Access to print*	−0.17*				
3. Literacy‐related activities*	−0.12*	0.32*			
4. Parents reading habits*	−0.16*	0.36*	0.37*		
5. Child's interest*	−0.12*	0.26*	0.43*	0.26^**^	
6. Emergent literacy*	−0.27*	0.25*	0.24*	0.17*	0.36*

Note: *n* = 821;

* *p* < 0.001.

a Factor scores of confirmatory factor analysis.

Table 5 presents results of the hierarchical regression analysis to find out whether FR status is a unique predictor before and after adding control variables. FR status was entered in step 1, and the three HLE factors; the child's gender, interest in literacy and letters, months in kindergarten and vocabulary; and parental educational level were entered as control measures in the second step. The amount of explained variance (*R* ^2^) before and after including background variables is also presented to show how FR status, with and without controlling variables, predicts variation in children's emergent literacy. To adjust for multiple comparisons and reduce type I error, the Benjamini–Hochberg correction (Benjamini & Hochberg, 1995) was applied within the regression analysis. This correction adjusts the critical *p*‐value in a stepwise manner based on the number of significance tests included within a particular set of analyses. However, this correction did not change the significant results, and we report the original *p*‐values in Table 5.

**Table 5:** Hierarchical regression analyses, predicting emergent literacy at age 6 years before onset of reading instruction

Variable	Step 1			Step 2		
	*B*	*SE B*	*β*	*B*	*SE B*	*β*
Family risk status	−0.63*	0.08	−0.26	−0.40*	0.07	−0.17
Access to print^a^				0.08	0.05	0.05
Literacy‐related activities^a^				0.02	0.05	0.02
Parents' own reading habits^a^				0.05	0.04	0.04
Child's interest in Literacy and letters^a^				0.37*	0.06	0.20
Children's gender				0.26*	0.06	13
Months in kindergarten				0.06	0.04	0.05
Vocabulary				0.10*	0.01	0.34
Mother – low education^b^				−0.08	0.16	−0.02
Mother – medium education^b^				0.11	0.08	0.05
Father – low education^b^				−0.45*	0.15	−0.10
Father – medium education^b^				0.10	0.07	0.05
*R* ^2^	0.068			0.334		
*F* for change in *R* ^2^	*F*(1, 782) = 56.95, *p* < 0.001			*F*(11, 771) = 27.94, *p* < 0.001		

a Factor scores of confirmatory factor analysis.

b Mother – high education and father – high education used as reference groups for parental educational level.

* Significant at *p* < 0.001.

For parental educational level, we used mother – high education and father – high education as the reference groups because high level of education was the largest group among the three levels of education for both mothers and fathers. Parents' educational level was used as a proxy for socioeconomic status as previous research has shown that in Norway, where this study was undertaken, parental level of education is a stronger predictor of educational outcomes than parents' income (Løken, 2010).

The FR status based on the parents' self‐report of RD (entered in step 1) accounted for 6.8% of the variance in children's emergent literacy. After adding the background variables simultaneously in step 2, a total of 33.4% of the variation in children's emergent literacy was explained. Unsurprisingly, FR status, based on parents' self‐report of RD, was a significantly negative predictor for the children's emergent literacy while controlling for HLE; the child's gender, interest in literacy and letters, months in kindergarten and vocabulary; and parental level of education before the onset of reading instruction. The three HLE factors, number of months in kindergarten and parental levels of education did not significantly contribute to the model. Besides FR status, the child's gender, interest in literacy and letters and vocabulary were related significantly to emergent literacy (Table 5).

## Discussion

We examined whether FR status based on parents' self‐report of RD could contribute to their children's performance in emergent literacy before formal reading instruction. In particular, we investigated whether having both parents with RD puts the child at higher risk for lower emergent literacy compared with having only one parent with RD, which had not previously been empirically reported.

Our findings extend previous FR studies by demonstrating that parents' self‐report of RD can be used as a single measure to determine children at FR risk in a large, representative sample. Our results showed that FR children were significantly impaired compared with not‐FR children on all measures of emergent literacy, including letter knowledge, first phoneme isolation, blending, vocabulary, RAN, STM, word reading and spelling. These findings were similar to previous FR studies where parents' self‐report of RD was corroborated by detailed reading assessments (Carroll & Snowling, 2004; Elbro *et al*., 1998; Elbro & Petersen, 2004; Nash, Hulme, Gooch, & Snowling, 2013; Torppa *et al*., 2007; Torppa *et al*., 2012).

The most important and novel finding from our study was the significant group differences in emergent literacy within the group of FR children that were apparent even before the onset of reading instruction. As expected, children with two parents self‐reporting RD had significantly poorer emergent literacy than either children with only one parent self‐reporting RD or children with no parent self‐reporting RD. Group differences in emergent literacy were moderate to large in letter knowledge, first phoneme isolation and vocabulary in favour of FR‐one children compared with FR‐both children. Unsurprisingly, the largest group difference in emergent literacy was found between FR‐both children and not‐FR children. These results are in line with the findings from Wolff and Melngailis' study of literacy outcomes for children with a genetic history of dyslexia (1994). Wolff and Melngailis (1994) reported that children in families with two dyslexia‐affected members were not only at greater risk but also more severely impaired than children with only one dyslexia‐affected family member. The current findings clearly suggest group differences within the FR group in emergent literacy, leaving children with two parents reporting RD with the lowest emergent literacy and thus more likely to experience some difficulties when learning to read, even if they do not go on to develop RD per se.

We also found significant differences in children's vocabulary between not‐FR and FR groups although the effect size in our study was small (*d* = 0.28) compared with previous studies (*d* = 0.65) (Snowling & Melby‐Lervåg, 2016). We had therefore expected that both groups would perform less well than the not‐FR group in vocabulary. However, we found that children with one parent self‐reporting RD did not significantly differ from not‐FR children. Furthermore, children with both parents self‐reporting as RD showed large deficits in vocabulary compared with children with only one parent self‐reporting as RD (*d* = 0.71), and not surprisingly, they showed even larger deficits compared with not‐FR children (*d* = 0.87). To our knowledge, this is the first study to report such a large deficit in vocabulary for FR children with two parents self‐reporting as RD. This novel and important finding suggests that children with both parents self‐reporting RD are not only at greater risk for RD but also may show severe difficulties because they have a wider range of language difficulties (Carroll & Snowling, 2004; Snowling & Hulme, 2012). Another possible explanation is that deficits in one area (e.g. vocabulary) may act as barriers to progress in another area (e.g. phonological processing skills). For example, according to Walley, (1993), children's vocabulary growth during the preschool years is critical for the development of their phonological representations from holistic or undifferentiated words to segmental forms. This lexical restructuring could be due to the growth of children's vocabulary through experience with spoken language and exposure to print. Interestingly, not only did FR children with two parents self‐reporting as RD score lower in vocabulary than FR‐one and not‐FR groups, but also their parents reported poorer HLE compared with the other groups, especially for the component of children's access to print.

Another important finding in the current study was the differences in HLE components between not‐FR and FR groups. Unsurprisingly, the not‐FR families reported a better overall HLE than FR children, and large group differences were found for access to print (*d* = 1.01), literacy‐related activities (*d* = 0.53) and parents' own reading habits (*d* = 0.79). These results were both similar and different to previous studies looking at FR and components of HLE. In contrast to the Finnish Jyväskylä study (Torppa *et al*., 2007), but more similar to research in English (Dilnot *et al*., 2017; Hamilton *et al*., 2016; Scarborough, 1991), we found significant group differences in the access to print factor between not‐FR and FR groups. Specifically, in the current study, parents in the FR group reported fewer children's books in the household than in the not‐FR group, while in the Finnish study, no significant differences were reported for this item. Furthermore, when both parents reported having RD (FR‐both), they tended to select the minimum number of books on the scale. However, the significant group difference we found between not‐FR and FR groups in the ‘parents own reading habits’ factor was compatible with the results of both the Finnish Jyväskylä study and HLE research in English. These results suggest that parents who report having RD are less active readers and, as a result, provide fewer incidences of positive reading models to their children, especially when both parents report RD. In contrast to the Finnish studies, our data suggest that parents in FR groups report less frequent literacy‐related activities at home compared with not‐FR group, and this effect held for both FR‐one and FR‐both groups. A likely explanation for the disparity in these results is the lack of significant differences in parental educational level between the FR and not‐FR groups in the Finnish studies, whereas in the present study and the English context, the parental educational levels were significantly lower in the FR group compared with the not‐FR group. The group differences in parental educational levels are not surprising because parental educational levels are typically reported as being lower in FR families (Snowling & Melby‐Lervåg, 2016).

Overall, these findings add to the understanding of the impact of parental history of RD on HLE by showing that not‐FR families provide the richest literacy environment for their children through positive reading models, literacy‐related activities and access to print material than families with at least one parent reporting RD, and the impact is especially severe when both parents report RD. One possible explanation for such differences in the HLE is the combination of influences from both FR (here, parents self‐reporting of RD) and parental low level of education in FR families. Because *FR status* is a possible reason for the lower level of parental education in the group of FR children, it could be argued that FR can have a direct negative impact and/or indirect one through parental level of education on the components of HLE.

Moreover, we investigated group differences in the different components of HLE within the FR group, which has not been previously explored. Our data suggest group‐level differences within FR groups in some components of HLE (especially in access to print, parental positive model of reading and some items of literacy‐related activities like frequency of shared reading).

Equally important, we investigated children's interest in literacy and letters before formal reading instruction. The FR children tended to show less interest in literacy and letters than not‐FR children (*d* = 0.25), according to their parents, which was not reported in the Finnish studies (Torppa *et al*., 2006, 2007). Parents in our study reported about their children's literacy interest by answering two questions about (1) reading interest and (2) interest in letters. Interestingly, no significant group effect was found for the item ‘my child often asks to be read to’; however, there was a significant group effect for ‘the child's interest in letters. This suggests that FR children are interested in shared reading as much as not‐FR children before formal reading instruction, which is more compatible with the results from the Finnish study. Nevertheless, it seems likely that FR children are not enthusiasts of letters and sounds as much as not‐FR children before formal reading instruction. These subtle yet important differences in children's reading interests should be taken into account in future research.

As expected, FR status was a unique predictor of emergent literacy before the onset of reading, even after controlling for background variables including the HLE and parental level of education. Besides FR status, children's gender, their interest in literacy and letters and vocabulary were also significant predictors of emergent literacy. It is notable that none of the HLE factors was a significant predictor, whereas it was assumed that the active HLE factor (literacy‐related activities) would be a unique contributor when these activities and the passive factor (access to print) are included simultaneously (Burgess, Hecht, & Lonigan, 2002).

Among variables for parental education, we found only a significant negative association between father's low education and children's emergent literacy, while this association was absent for mothers with low‐education level. A possible reason for this is that in our data the fathers' level of education was significantly lower than the mothers' level of education; however, children's emergent literacy and parental low‐education level showed similar strengths of associations (~0.24 for mothers and ~0.18 for fathers). In addition, FR status (parents' self‐report of RD) was significantly, but negatively, correlated to their children's emergent literacy (approximately −0.26). These correlations were lower than the correlations reported by van Bergen *et al*. (2012) for children and parents' literacy skills, because they assessed parents' reading fluency.

Finally, it is noteworthy that parental reports of children's interest in literacy and letters were found to be related to their concurrent emergent literacy. This finding replicated previous research (Frijters, Barron, & Brunello, 2000) that suggested children's literacy interest may be a key driver of the relationship between the HLE and emergent literacy. However, the direction of causality of this relationship is not clear, because all constructs were measured concurrently.

### Implication of the Findings for Parents, Teachers and Early Educational Settings

Overall, the results of this study indicate a substantial influence of parental history of RD on children's emergent literacy performance before reading onset and some components of the HLE. The causal mechanisms underlying the development of children's emergent literacy, however, cannot be inferred from a correlational design such as the present study. It is possible that the self‐report of RD is indicating parental confidence in reading, rather than their actual reading skills/difficulties, which is also likely to impact on HLE. Nonetheless, what is important to note is the association between parents' self‐reporting of RD, poor HLE and their children's low emergent literacy.

Together, our findings have important implications for practitioners and teachers working with preschool children and first‐grade students. Although the language context of the present study is Norwegian, our findings can be generalized to other contexts. Snowling and Melby‐Lervåg (2016), in their meta‐analysis study, reported that FR children, regardless of the language context, perform significantly poorer in emergent literacy skills than not‐FR children at preschool ages, which in turn puts FR children at greater risk for RD. They highlight that these findings, which included studies in both English and Finnish contexts, are consistent across languages, and the effect of orthography is only marginal. The current results in Norwegian, which is a semi‐transparent orthography that is more regular than English and less regular than Finnish, are consistent with their findings.

The principal implication is the value of screening for possible risk of RD in children with the simple but valid tool ‘parents' self‐report of RD’ in preschool years. Our findings suggest that parents will report having experienced reading and writing problems if they found them challenging subjects at school. Furthermore, the present study clearly demonstrates that if the parents experienced RD, their children are more likely to experience difficulties in developing emergent literacy at preschool ages and that the risk of difficulties in emergent literacy is higher when both parents have a history of RD. The parents' self‐report of RD is not sufficient to know whether a child will go on to develop RD, but it provides a good starting point for further assessment or attention for preschool children.

Although our data do not allow us to draw inferences about causation, these findings suggest that the early speech and language development of children at FR risk of RD should be monitored closely, especially when both parents have reported experiencing reading and writing problems. Our data clearly showed a broad range of language difficulties, particularly in vocabulary skills of FR children when both parents report having RD.

Another implication concerns reading‐related habits and activities in the home. The current study found that the HLE for families where the parents self‐reported RD was not as rich as in not‐FR families. In addition, exposure to print material is less frequent when both parents self‐report RD. Parents, especially those who self‐report RD, should be advised about the crucial role of the HLE in the development of their children's emergent literacy and possibly later reading skills. This study indicates that families where both parents self‐report RD have the fewest number of children's books at home, fewer than families with only one parent self‐reporting RD, who, in turn, reported having fewer books than families with no self‐report of RD. Previous research indicated that a richer HLE is associated with enhanced vocabulary skills in early and middle childhood. Niklas and Schneider (2015) found that HLE interventions can have an impact on home learning environments and children's language development including their vocabulary. Parents need to know that they can support their children's emergent literacy by providing them with better access to print and more shared‐reading activities.

## Limitation and Future Study

The present study has some potential limitations regarding the measure of HLE. Based on previous research, the HLE measure was a questionnaire designed to capture the home environment from the aspect of the child's access to print, reading‐related activities in the home, parents' own reading habits and children's interest in literacy. First, similar to most previous research, the HLE questionnaire was the only measure and there were no data on parent–child interactions. The present study was a quantitative large‐scale design; however, future qualitative studies would shed more light on this matter. Second, parents' potential reading problems and/or their confidence in reading, especially in the group of parents who self‐reported RD, could raise some concerns regarding their ability to fully comprehend the questionnaire. In order to mitigate the impact of this, we used a specially designed questionnaire containing short, simple, multiple‐choice questions for parents to answer at home at their own pace. Third, children's oral language skills were assessed through the vocabulary test, and we did not include a measure of the quality or quantity of spoken language in the home environment as it was not the focus of the current HLE measure. Future studies should assess the possible impact of the quality and quantity of spoken language in the home on children's literacy‐related skills such as vocabulary.

## Conclusions

Despite some limitations, this study provides clear evidence of difficulties across a range of emergent literacy skills in FR children. The present study adds to the literature on FR of RD by using parents' self‐report of RD as a single measure for identifying group differences in emergent literacy before the onset of reading instruction. Our findings show impaired emergent literacy and poor HLE in children with parental history of RD in line with previous studies on FR of RD, which included corroborating measures along with parents' self‐report of RD to confirm FR status. Furthermore, we found that FR children may also experience less exposure to literacy material in the home compared with not‐FR children. The parents of FR children also reported that their children were less interested in letters. These findings suggest that children's experience in their home environments and their interest in letters may not be independent of FR factors. Overall, given the importance of early identification, this study highlights the value of parents' self‐report of RD as a single measure, where parental self‐report of RD is evidently associated with differences in HLE and in turn correlated with children's emergent literacy. Our findings have critical implications for schools, teachers and early years' settings.

Finally yet importantly, a novel aspect of this study was to investigate group differences in emergent literacy within the FR group, which has not been explored in past FR studies. Children with two parents self‐reporting RD showed a broad range of language difficulties compared with children with only one parent self‐reporting RD and children with no such risk. This study suggests that parents' self‐report of RD can demonstrate FR of RD as early as preschool.
